# Adaptation and validation of the Polish version of the Quality from Patient Perspective-Intrapartal (QPP-I) questionnaire to assess childbirth care

**DOI:** 10.18332/ejm/201472

**Published:** 2025-05-12

**Authors:** Julia Nawrot, Dorota Matuszyk, Aneta Suder, Agnieszka Bień, Violetta Skrzypulec-Plinta, Agnieszka Gniadek

**Affiliations:** 1 Institute of Nursing and Midwifery, Jagiellonian University, Cracow, Poland; 2 Institute of Physiotherapy, Jagiellonian University, Cracow, Poland; 3 Chair of Obstetrics Development, Faculty of Health Sciences, Medical University of Lublin, Lublin, Poland; 4 Department of Women’s Health, Faculty of Health Sciences, Medical University of Silesia, Katowice, Poland

**Keywords:** quality of care, perinatal care, childbirth experience, patient satisfaction, midwifery, questionnaire

## Abstract

**INTRODUCTION:**

As midwifery shifts to a holistic, women-centered model, assessing care quality from the patient's perspective is crucial. The lack of standardized tools in Poland forces reliance on invalidated *ad hoc* measures. This study bridges the gap by translating and validating the QPP-I questionnaire.

**METHODS:**

The Polish QPP-I was adapted and validated through a cross-cultural study. A pilot (25 women) and a multicenter study (153 women) were conducted 2–3 days postpartum, with a test-retest after 2–4 weeks. Convenience sampling was used, with data collected via online and paper questionnaires. The pilot ran in late 2019, and the main study (February 2020–March 2021) spanned five maternity wards. Internal consistency, test-retest reliability, and criterion relevance were analyzed.

**RESULTS:**

The questionnaire was well-received by the target group, requiring minimal cultural adaptation. QPP-I PL demonstrated high internal consistency (α=0.935 for the 1st testing, α=0.95 for the test-retest) and good validity (mean Kendall W=0.65). Reliability was assessed using Cronbach’s alpha. Most items showed good reliability (α >0.70). The Perceived Reality (PR) subscale had high reliability (α=0.90), while the Subjective Importance (SI) subscale reached α=0.93, confirming the appropriateness of all items. However, indicators related to participation in decision-making and midwifery attendance showed poor internal consistency. The mean alpha coefficient in the test-retest further supported good reliability (α=0.65).

**CONCLUSIONS:**

The Polish version of the QPP-I questionnaire demonstrates good validity and reliability for assessing the quality of perinatal care from the patient's perspective. The questionnaire reflects the Polish perinatal care context while maintaining the original tool's integrity.

## INTRODUCTION

Improving the quality of perinatal care continues to be a significant challenge both regionally and globally^1,2^. Evidence-based practice (EBP), essential for delivering high-quality care, goes beyond implementing the latest research or clinical guidelines. It also involves recognizing and integrating patients’ needs, preferences, and experiences^3^. Modern approaches argue that perinatal care cannot be considered high-quality if the woman is not satisfied with the care provided. Pregnancy and natural childbirth are, by definition, physiological processes. Except in complications, they do not require complex medical interventions, which is also important from the perspective of those studying the non-medical aspects of perinatal care. This assumption may shift women’s attention to other non-medical aspects of care, such as a sense of intimacy, the approach of the medical staff, psychological and emotional support, or trust. Quality of care is intrinsically linked to women’s satisfaction, as studies have shown a strong correlation between a woman’s positive experience during childbirth and better clinical outcomes for both mother and baby during the postpartum period and through the first years of a child’s life^4,5^. Moreover, a positive childbirth experience can impact a woman’s psychological wellbeing and perception of future pregnancies.

Despite various improvements in the Polish healthcare system over the last few decades, further enhancement in perinatal care quality remains essential^6^. One of the barriers to achieving this is the absence of a valid and reliable tool that adequately measures birth satisfaction and quality of care from the woman’s perspective. To address this, we aimed to perform a cross-cultural adaptation and validate the widely recognized Quality from Patient Perspective-Intrapartal (QPP-I) questionnaire^7^ for use in the Polish context. This validated tool will allow for a more structured and reliable assessment of the quality of perinatal care, supporting both clinical practice and future research efforts to enhance maternal care.

## METHODS

### Study design and setting

The study was cross-sectional with an adaptation, validation, and reliability testing component. The study was designed based on a five-step adaptation process. Firstly, permission to use the questionnaire and to participate in the procedure as a consultant for verifying the accuracy of the translation of the original version was obtained from the authors of the QPP-I questionnaire. Secondly, we performed the translation process. Two independent translations were made from Swedish to Polish (forward translations). Both translators were Polish, but just one was a specialist in medical translations. A group of 12 experts in midwifery, nursing, health sciences, and linguistics analyzed both versions, reviewed all differences, and gave their comments. Afterward, the next version was prepared and translated back into Swedish. This version was consulted with the authors of QPP-I and then translated into Polish. The next step was pilot testing of the preliminary version.

A translation-back translation procedure was carried out to prepare the Polish version of the questionnaire. Then, after obtaining expert opinions, modifications were made to the content of the questions to adapt the questionnaire to the currently prevailing standards of hospital care in the organizational and technical sphere in Poland. The authors’ validated English-language version of the tool was additionally used in this process. The use of auxiliary language versions other than the original one can contribute to the detection of an error in an already existing translation^8^. The next step was back-translation and consultation with the authors of the original version. A high level of equivalence was found between the two language versions. Most of the authors’ suggestions were included. The final translation was evaluated in a pilot study in one of the hospitals in Kraków, Poland. The questionnaire was completed by 24 women who responded to the survey translated into Polish. Just one of the questions seems to be grammatically incorrect. All the respondents agreed that the questions were clear and intelligible. The graphical side of the questionnaire was also prepared to be similar to the original version.

### Data sources

The validation study had a multicenter character and was conducted in five Polish hospitals in Malopolskie, Podkarpackie, and Zachodniopomorskie voivodeships, including two university hospitals, one specialist hospital of the third referral level, one second referral level ward, and one first referral level ward. The study was conducted between February 2020 and March 2021 with the pilot study taking place in February 2020; data collection was carried out between March 2020 and March 2021 with test-retest at 2–4 weeks after the initial completion of the questionnaire. Data were obtained from a cross-sectional validation study using a self-administered questionnaire completed by participants. Due to the ongoing COVID-19 pandemic and the restrictions during the lockdown, the collection of questionnaires in Poland was temporarily suspended.

### Participants

Given the relatively high cultural homogeneity of the Polish population, including limited religious diversity, a low percentage of immigrants, and national minorities, five hospital wards were selected for the study, with diversity in terms of location and level of referral care. Participants were recruited using a convenience sampling method from hospitals and maternity clinics in Poland, with eligibility criteria including age ≥18 years, proficiency in Polish both spoken and written, early postpartum after vaginal birth or a cesarian section, hospitalized in the postnatal ward, and consent to participate in the study. The following criteria were used to exclude participants from the study: women aged <18 years, with a status after miscarriage or stillbirth, with a health condition requiring intensive medical interventions, impaired consciousness or disorientation during the hospital stay, and women who could not speak or write Polish. The pilot study included results from 25 women. The final sample of the validation group included 153 women. Both groups were women hospitalized after childbirth who agreed to participate in the study on their 2nd to 3rd day postpartum. Each researcher received recruitment instructions, a paper version of the scale, and a consent form template. A set of unique, computer-generated alphanumeric tokens was prepared as access codes for the online survey. To carry out a test-retest, we also asked participants if they were interested in filling in again the questionnaire online. The invitation with a link to the platform was sent by a text message. The electronic version of the tool was implemented in the Jagiellonian University Survey Research System on the LimeSurvey server (Version 3.25.7+210113). Before deployment, it was tested for usability and navigation ease.

### Measurement

Quality from Patient Perspective - Intrapartal specific questionnaire is a self-completion questionnaire from Sweden. It was designed by Wilde-Larsson et al.^7^ in 2010 based on a general measure of quality called Quality from the Patient’s Perspective (QPP)^7^. Intrapartal version is one of the available versions of the QPP scale in Sweden^8^. It has also been translated, validated, and used in other countries such as Spain^9^. Numerous studies show that the QPP-I offers good psychometric properties^9-11^. The QPP-I scale consists of three main parts, which include 62 items in total, from which 40 are classified in four main dimensions of the quality of care: medical-technical skills, physical-technical conditions, focus on safety, and the sociocultural environment. Respondents evaluate satisfaction in four different areas: medical competencies, medical and technical factors, technical conditions, infrastructure, individualized approach (identity-oriented approach), and social and cultural aspects of care received. The first part includes questions about demographics and medical information like parity, length of pregnancy, and way of birth. The next part contains questions about care during labor, birth, and the early postpartum period, such as receiving information, respectful care, support, the atmosphere, and the birth environment. The scale is divided into two subscales – perceived reality (PR) and subjective importance (SI). The participants choose one of the options on both subscales. The scores for each item are from 1 to 4. The higher the score for each item, the greater the satisfaction. There is also a ‘non-applicable’ option, for example, for women who had a cesarian section. The last part consists of questions about feeling safe during labor, being proud of giving birth, and current physical and psychological health. It also contains a free-text option for women, which may be used to deepen knowledge about women’s birth experiences. The result of quantitative questions is calculated with an action index, which presents the areas of care in four categories: deficient quality, balance low quality, balance high quality, and excess quality of care. The cut-offs for the four described categories are presented in Table 1. It is questionnaire is administrated at discharge.

**Table 1:** The cut-offs for the quality categories for Action Index calculation in QPP-I questionnaire

*Category*	*Patient responses to the questions on the PR subscale*	*Corresponding responses on the SI subscale*
**Deficient quality**	Partly agree	Highest importance
	Partly agree	Highest importance
	Don’t agree	Highest importance
	Don’t agree	High importance
	Mostly agree	Highest importance
	Partly agree	High importance
	Don’t agree	Some importance
**Balance low quality**	Partly agree	Some importance
	Don’t agree	Little or no importance
**Balance high quality**	Mostly agree	High importance
	Mostly agree	Highest importance
**Excess quality of care**	Fully agree	High importance
	Mostly agree	Some importance
	Partly agree	Little or no importance
	Fully agree	Little or no importance
	Mostly agree	Little or no importance
	Fully agree	Little or no importance

PR: perceived reality. SI: subjective importance.

### Statistical analysis

Statistical analysis was performed using the IBM SPSS Statistics 26 package. Basic descriptive statistics were analyzed along with the Shapiro-Wilk test. Cronbach’s alpha was calculated for internal consistency, with coefficient values >0.7 considered satisfactory. The test-retest method and Wilcoxon test were used to assess reliability. Test validity was analyzed using Kendall’s W coefficient of concordance (W≥0.70 indicating high validity) and Spearman’s r correlation (r≥0.4 indicating a relationship). Results were considered statistically significant at p<0.05.

### Ethical considerations

The Bioethics Committee of Jagiellonian University approved the study, Kraków (approval no. 1073.6230.36.2017, approval date: 20 June 2017). Respondents were informed that participation was voluntary, study results were anonymous and to be used exclusively for research purposes, and they provided informed consent.

## RESULTS

The study group included 153 women aged 21–43 years (mean age=31.22 years; SD=4.50). The vast majority of the sample lived with their husband or partner (92.8%) and had a higher education qualification (81.0%). The study group was divided into women having their first child (50.3%) and subsequent births (49.7%). Most respondents gave birth between 37 and 42 weeks’ gestation (90.8%). A majority of the study group had a vaginal birth (63.4%). Complete participant descriptive statistics are found in Table 2.

**Table 2:** Characteristics of study group, cross-sectional multicenter study of postpartum women, Poland, 2020–2021 (N=153)

*Characteristics*	*Categories*	*n*	*%*
**Age** (years), mean (SD)		31.22 (4.50)	
**Civil status**	Cohabitating	149	97.4
	Single	4	2.6
**Education level**	Elementary	2	1.3
	High school	27	17.6
	University	124	81.0
**Which birth**	First	77	50.3
	Second or higher	76	49.7
**Week of pregnancy when the baby was born**	Before 37 weeks	14	9.2
	Between 37 and 42 weeks	139	90.8
**Way of birth**	Vaginal birth	97	63.4
	Vaginal delivery with VE	2	1.3
	Planned CS	39	25.5
	Emergency CS	15	9.8

VE: vacuum extractor. CS: cesarian section.

To check the distribution of the quantitative variables, basic descriptive statistics were calculated with the Shapiro-Wilk test examining the normality of the distribution. For the purpose of calculation and interpretation, responses were recorded in ascending order, where 1 represents the lowest response on the scale and four the highest. The result of the Shapiro-Wilk test proved statistically significant. This means that the distributions deviate significantly from the normal distribution. Moreover, the skewness of the distribution of most variables exceeds the absolute value of 1, which means that their distributions are asymmetric to a significant degree. Therefore, further analyses were based on non-parametric tests.

To analyze the reliability of the questionnaire, Cronbach’s alpha coefficient was calculated (Table 3). Most items in both measures had good reliability (α>0.70). In the Perceived Reality subscale (PR), reliability was 0.90. For the Subjective Importance (SI), reliability reached 0.93, suggesting that all items were appropriate to measure. There was poor internal consistency for indicators on the PR and SI subscales for participation in decision-making and midwifery attendance on the SI subscale. The mean value of the alpha coefficient for both subscales in the test-retest measurement proved good reliability as well. However, the results showed a mixed range of Cronbach’s α values (Measurement 1: 0.45–0.84 for the PR subscale and 0.31–0.86 for the SI scale; Measurement 2: 0.38–0.93 for the PR subscale and 0.48–0.92 for the SI scale).

**Table 3:** Cronbach’s α results for perceived reality and subjective importance subscales for QPP-I scale, cross-sectional multicenter study of postpartum women, Poland, 2020–2021 (N=153)

*Characteristics*	*Cronbach’s α*	*Number of items*
**Perceived reality (PR)**		
Information – before/after procedure	0.754	2
Information – self-care	0.490	2
Commitment, empathy and respect (midwives)	0.781	5
Midwives present during labor	0.749	2
Medical care and pain relief	0.824	8
Participation in decision making	0.459	2
Commitment, empathy and respect (other staff)	0.773	3
Commitment, empathy and respect (doctors)	0.787	3
Partner/significant other	0.783	6
Care equipment/atmosphere	0.848	5
General	0.907	
**Subjective importance (SI)**		
Information – before/after procedure	0.866	2
Information – self-care	0.572	2
Commitment, empathy and respect (midwives)	0.798	5
Midwives present during labor	0.416	2
Medical care and pain relief	0.825	8
Participation in decision making	0.316	2
Commitment, empathy and respect (other staff)	0.829	3
Commitment, empathy and respect (doctors)	0.854	3
Partner/significant other	0.744	6
Care equipment/atmosphere	0.714	5
General	0.889	
**Total for PR and SI subscales**	0.935	

Removing items with the lowest α coefficient did not bring significant differences in the results. Thus, it was decided to leave the original layout of the questionnaire with the few low-reliability values. In the test-retest results, there was relatively slight variation in the responses of individual respondents to individual questions. Still, at the same time, the responses of the entire study group to the same questions were relatively different.

To assess the reliability and repeatability of the test, it was checked whether the results of the individual indicators from the first measurement differed from the results of the second measurement (test-retest). The results of the analysis are presented in Table 4.

**Table 4:** Test-retest analysis, cross-sectional multicenter study of postpartum women, Poland, 2020–2021 (N=153)

*Characteristics*	*Measurement 1*		*Measurement 2*		Statistical analysis*		
	*Mean*	*SD*	*Mean*	*SD*	*Z*	*p*	*r*
**Perceived reality (PR)**							
Information – before/after procedure	3.53	0.65	3.50	0.55	-0.10	0.917	0.01
Information – self-care	3.63	0.46	3.52	0.65	-0.11	0.913	0.01
Commitment, empathy and respect (midwives)	3.66	0.38	3.62	0.52	-0.34	0.735	0.03
Midwives present during labor	3.85	0.38	3.63	0.46	-0.28	0.783	0.04
Medical care and pain relief	3.66	0.43	3.85	0.38	-1.72	0.086	0.17
Participation in decision making	3.47	0.59	3.45	0.56	-0.10	0.917	0.01
Commitment, empathy and respect (other staff)	3.69	0.45	3.68	0.45	-0.31	0.756	0.03
Commitment, empathy and respect (doctors)	3.57	0.56	3.53	0.57	-0.36	0.722	0.04
Partner/significant other	3.86	0.38	3.86	0.36	-0.36	0.722	0.05
Care equipment/atmosphere	3.74	0.47	3.70	0.47	-0.64	0.523	0.06
**Subjective importance (SI)**							
Information – before/after procedure	3.71	0.46	3.78	0.44	-1.20	0.229	0.12
Information – self-care	3.63	0.37	3.63	0.39	-0.13	0.901	0.01
Commitment, empathy and respect (midwives)	3.78	0.27	3.80	0.34	-0.65	0.518	0.07
Midwives present during labor	3.85	0.31	3.89	0.30	-0.14	0.890	0.02
Medical care and pain relief	3.79	0.29	3.80	0.27	-0.12	0.902	0.01
Participation in decision making	3.71	0.40	3.74	0.41	-0.29	0.768	0.03
Commitment, empathy and respect (other staff)	3.73	0.55	3.78	0.50	-0.67	0.505	0.07
Commitment, empathy and respect (doctors)	3.75	0.49	3.75	0.49	-0.09	0.931	<0.01
Partner/significant other	3.85	0.30	3.84	0.30	<0.01	1.000	<0.01
Care equipment/atmosphere	3.50	0.55	3.44	0.55	-0.44	0.659	0.04

r: effect-size indicator. Measurement 1: first questionnaire completion. Measurement 2: repeated questionnaire completion.

* Wilcoxon test.

The analysis did not reveal any statistically significant differences between the results of individual indicators in the first and second measurements. Regardless of the timing of the questionnaire completion, the subjects demonstrated a similar level of assessment for various aspects of their stay in the maternity ward. Time did not differentiate the levels of individual indicators, which also indicates the consistency of the questionnaire.

For the validity study, Kendall’s coefficient of concordance (W) was used (Table 5).

**Table 5:** Kendall’s coefficient of concordance (W), cross-sectional multicenter study of postpartum women, Poland, 2020–2021 (N=153)

*Factor*	*Measurement 1*			*Measurement 2*			*Statistical analysis*	
	*Median*	*Q1*	*Q3*	*Median*	*Q1*	*Q3*	*W*	*p*
**Perceived reality (PR)**								
Information – before/after procedure	4.00	3.50	4.00	4.00	3.00	4.00	0.68	**0.046**
Information – self-care	4.00	3.50	4.00	4.00	3.00	4.00	0.74	**0.021**
Commitment, empathy and respect (midwives)	4.00	3.75	4.00	3.80	3.40	4.00	0.77	**0.009**
Midwives present during labor	4.00	4.00	4.00	4.00	4.00	4.00	0.67	0.132
Medical care and pain relief	3.75	3.33	4.00	3.78	3.33	4.00	0.68	0.051
Participation in decision making	3.50	3.00	4.00	3.50	3.00	4.00	0.48	0.562
Commitment, empathy and respect (other staff)	4.00	3.33	4.00	4.00	3.33	4.00	0.59	0.183
Commitment, empathy and respect (doctors)	4.00	3.33	4.00	4.00	3.00	4.00	0.50	0.457
Partner/significant other	4.00	3.80	4.00	4.00	3.82	4.00	0.68	0.095
Care equipment/atmosphere	4.00	3.50	4.00	4.00	3.50	4.00	0.58	0.206
**Subjective importance (SI)**								
Information – before/after procedure	4.00	3.00	4.00	4.00	3.88	4.00	0.81	**0.004**
Information – self-care	3.50	3.50	4.00	3.50	3.50	4.00	0.58	0.224
Commitment, empathy and respect (midwives)	4.00	3.68	4.00	4.00	3.60	4.00	0.68	**0.050**
Midwives present during labor	4.00	3.50	4.00	4.00	4.00	4.00	0.65	0.146
Medical care and pain relief	3.88	3.67	4.00	3.88	3.63	4.00	0.63	0.107
Participation in decision making	4.00	3.50	4.00	4.00	3.50	4.00	0.70	**0.033**
Commitment, empathy and respect (other staff)	4.00	3.67	4.00	4.00	4.00	4.00	0.74	**0.020**
Commitment, empathy and respect (doctors)	4.00	3.67	4.00	4.00	3.92	4.00	0.71	**0.030**
Partner/significant other	4.00	3.50	4.00	4.00	3.67	4.00	0.62	0.161
Care equipment/atmosphere	3.57	3.00	4.00	3.46	3.00	4.00	0.61	0.141
**General**							**0.65**	**0.04**

Q1: first quartile. Q3: third quartile.

The analysis showed that the indicators for most of the items had a good level of agreement (0.48<W<0.81). The same subjects rated the different aspects of their stay in the maternity ward similarly at discharge and after a certain period. The lowest Kendall score (W=0.48) was for participation in decision-making (perceived reality subscale) and provision of information for self-care (subjective importance subscale) (W=0.58). The highest score was achieved by the perceived reality subscale indicator ‘midwives’ attitude towards the patient’ (W=0.77) and on the subjective importance subscale ‘providing information on medical procedures’ (W=0.81). The mean value of all coefficients was 0.65, so the questionnaire was reliable.

## DISCUSSION

The main goal of this study was to prepare the Polish version of Quality from Patient Perspective- Intrapartal questionnaire. This instrument, as an easily administered, valid, and reliable scale, is supposed to evaluate quality of care during childbirth, labor and early postpartum period from the perspective of woman who gave birth. This scale, structured into four main dimensions of the quality of care: medical-technical skills, physical-technical conditions with 40 items, focus on safety, and the sociocultural environment, appears to be an effective tool for the studied population for assessing quality from women’s perspective. The results suggest that it can be effectively used in research and clinical settings to evaluate the quality of perinatal care and identify areas for improvement, as positive birth experiences play a crucial role in the well-being and long-term health of both women and their newborns.

Central to The World Health Organization’s Intrapartum Care for a Positive Childbirth Experience guidelines and recommendations, is the concept of Respectful Maternity Care (RMC), which recognizes women’s lived and experiential knowledge as an essential aspect of woman-centered care^12,13^. However, relatively few measures have been developed and appropriately tested in Poland, and there is a noticeable lack of adequate instruments to assess this important aspect of maternity care. In other countries, the QPP-I questionnaire is one of many tools used to measure satisfaction with care during childbirth^14-17^.

As van Campen et al.^18^ emphasize in a study on patient satisfaction evaluation, in order to properly measure patient satisfaction, the questionnaire should be based on a well-established theoretical foundation, consider basic criteria of quality of care, and be tested for reliability and validity, and can be used in a large population study. The QPP-I fulfills all these criteria. It was also tested before for its psychometric properties, such as content validity, internal consistency, and structural validity, with good results^7^. The analysis of the psychometric properties of the Polish version of the questionnaire also showed that the tool could be considered reliable with a mean α >0.80 for both subscales. However, the results of this study showed a mixed range of Cronbach’s α values (0.45–0.84 for the Perceived Reality subscale and 0.31–0.86 for the Subjective Importance scale). Most of the indicators obtained an α >0.7, indicating good scale reliability. The lowest score was obtained for the indicators relating to a woman’s participation in decision-making (for the Perceived Reality subscale α=0.45, and for the Subjective Importance scale α=0.31). It was also shown that to improve the low values of this indicators, removing questions would not bring significant differences in the results, so it was decided to leave the original layout of the questionnaire with few low reliability values. In their evaluation of the original version, Wilde-Larsson et al.^7^ also note that the reliability value of the entire scale is mixed (range for the Perceived Reality subscale α=0.50–0.92; range for the Subjective Importance subscale α=0.49–0.93). Factors with lower reliability were ‘medical-technical factors’ with α values of 0.50 and 0.58 for the PR and SI scales, respectively, and ‘midwives-to-patients approach’ with α values of 0.49 and 0.57 for the PR and SI scales, respectively. Similar statements were made by Donate-Manzanares et al.^9^ in assessing the properties of the Spanish-language version of the questionnaire. They prove that the tool has valid reliability values and that most, except for three indicators, obtained an α value >0.7. However, it is also impossible to strictly compare these results with this adaptation of QPP-I because most of them present results of confirmatory factor analysis, which is a different method. Sawyer et al.^10^ emphasize that the lower values for the mentioned indicators are due to the smaller number of test items, which is also reflected in the results of the Polish version of the questionnaire. In a 2017 systematic review, Alfaro Blazquez et al.^17^ detail it among 17 recent and theoretically studies on this topic. Twelve of these are original questionnaire adaptations of an already existing tool. Most of them were constructed in Europe. These tools are characterized by differences in the selection criteria of the study group, the time of completion, the scale construction, and the number of subscales in the questionnaires. Alfaro Blazquez et al.^17^ highlight that three tools analyzed, including the QPP-I, do not formulate specific inclusion criteria for the study group^.^ The authors of the QPP-I only take language skills and gender as inclusion criteria. This makes it possible to use the questionnaire both for women who have had a natural birth and those who have had a cesarean section. As Ängeby and Ternström^13^ demonstrate, the free-text option allows the questionnaire to be used in qualitative studies to refine the quality of care.

### Strengths and limitations

The main strength of this study lies in providing a reliable tool for healthcare practitioners and public health professionals, offering immediate applicability for quality-of-care assessments. It contributes to the field by introducing a validated Polish language version of the QPP-I questionnaire, a well-established and rigorously constructed instrument. Another strength of our study is its broad geographical scope, covering three large provinces in Poland. This provides a diverse sample of participants and allows for better generalization of the results across the country, reflecting regional differences in perinatal care experiences. This study has limitations, including the potential for response bias and the relatively short follow-up period. Additionally, the test-retest reliability was assessed within a limited timeframe of 2–4 weeks, which may not fully capture the tool’s stability over longer periods. Another limitation is a relatively small sample size, which may affect the generalizability of the findings. Moreover, the use of convenience sampling introduces a risk of selection bias, as the sample may not be representative of the broader population, potentially limiting our findings’ generalizability. Future research involving larger and more diverse populations is recommended to further validate the tool and enhance its applicability across different contexts and demographic groups. Additionally, longitudinal studies could help assess the tool’s sensitivity to changes in care quality over time. When analyzing the limitations of this study, the racial and cultural homogeneity of the group should also be considered.

## CONCLUSIONS

The validation of the QPP-I questionnaire offers a valuable tool for accurately measuring respectful maternity care in Poland. It demonstrates good validity and reliability for assessing the quality of perinatal care from the patient’s perspective. The internal consistency indicators, with Cronbach’s alpha values mostly above 0.70, confirm its reliability in measuring perceived reality and subjective significance in the context of childbirth and early postpartum care. The test-retest analysis indicates that the questionnaire provides consistent results over time, with no statistically significant differences between the two sets of measurements. This suggests that the tool can reliably assess the quality of care as perceived by women shortly after childbirth and later. The questionnaire reflects the specific organizational and cultural context of Polish perinatal care while maintaining the integrity of the original tool. The study included expert consultations and pilot tests, contributing to a high equivalence level between the original and adapted versions. As a future line of research expanding QPP-I usage to diverse healthcare settings and populations could provide a more comprehensive understanding of the determinants of high-quality care. Longitudinal studies are needed to assess the long-term impact of respectful maternity care on maternal satisfaction outcomes. This study contributes to the growing body of evidence on patient satisfaction and quality assessment tools, reinforcing the importance of integrating women’s perspectives into healthcare evaluations to drive meaningful improvements in perinatal care services.

## Data Availability

The data supporting this research are available from the authors on reasonable request.
